# Metabolic disorders and osteoarthritis: novel insights into pathological mechanisms and therapeutic strategies

**DOI:** 10.1093/lifemedi/lnag029

**Published:** 2026-07-15

**Authors:** Chunchun Xue, Wenyun Kui, Xiaofeng Li, Lingxing Li, Lilong Song, Dong Mao, John R Speakman, Ling Qin, Liping Tong, Di Chen

**Affiliations:** Shanghai Municipal Hospital of Traditional Chinese Medicine, Shanghai University of Traditional Chinese Medicine, Shanghai 200071, China; Department of Anesthesiology and Pain Rehabilitation, Cardiopulmonary and Critical Care Rehabilitation Center, Shanghai Yangzhi Rehabilitation Hospital, School of Medicine, Tongji University, Shanghai 201613, China; Shanghai Municipal Hospital of Traditional Chinese Medicine, Shanghai University of Traditional Chinese Medicine, Shanghai 200071, China; Shanghai Municipal Hospital of Traditional Chinese Medicine, Shanghai University of Traditional Chinese Medicine, Shanghai 200071, China; Shanghai Municipal Hospital of Traditional Chinese Medicine, Shanghai University of Traditional Chinese Medicine, Shanghai 200071, China; Orthopaedic Institute, Wuxi Ninth People’s Hospital Affiliated to Soochow University, Wuxi 214062, China; Center for Energy Metabolism and Reproduction, Shenzhen Institutes of Advanced Technology, Chinese Academy of Sciences, Shenzhen 518055, China; School of Biological Sciences, University of Aberdeen, Aberdeen AB24 3FX, United Kingdom; Musculoskeletal Research Laboratory of Department of Orthopaedics & Traumatology, Li Ka Shing Institute of Health Sciences, The Chinese University of Hong Kong, Hong Kong 999077, China; Center for AI-Driven Medical Research, Shenzhen Institutes of Advanced Technology, Chinese Academy of Sciences, Shenzhen 518055, China; Center for AI-Driven Medical Research, Shenzhen Institutes of Advanced Technology, Chinese Academy of Sciences, Shenzhen 518055, China; Faculty of Pharmaceutical Sciences, Shenzhen University of Advanced Technology, Shenzhen 518107, China

**Keywords:** osteoarthritis, metabolic disorder, obesity, pain, comorbidity

## Abstract

Metabolic disorders and osteoarthritis (OA) are chronic noncommunicable diseases with high global prevalence. Accumulating evidence underscores a strong association between metabolic illnesses and the onset and progression of the OA. This review systematically summarizes the current insights and mechanisms between metabolic disorders (obesity, hyperglycemia, dyslipidemia, hypertension, and gut microbiota dysbiosis) and OA from both epidemiology and molecular biology perspectives. Furthermore, we comprehensively elaborate on the latest therapeutic strategies for metabolic disease-related OA, emphasizing chronic inflammation and mitochondrial dysfunction as core pathophysiological bridges. Collectively, this review provides a theoretical and translational overview for the precise management of metabolic-related OA to facilitate new discoveries in chronic disease comorbidity prevention.

## Introduction

Osteoarthritis (OA) is a highly prevalent degenerative joint illness and a primary cause of physical impairment, impacting over 500 million individuals globally [1]. The prevalence exhibits a notable association with age: it affects 10%–20% of those aged 50 and beyond, increasing to approximately 50% for those aged 65 and above [2, 3]. The pathological features of OA encompass the gradual degeneration of articular cartilage, synovial inflammation, and the emergence of subchondral bone sclerosis [3, 4]. OA was primarily regarded as a result of “wear and tear” on the joints due to excessive activity and loading. Recent research suggests that the initiation and progression of OA are affected by a combination of metabolic factors, such as obesity, impaired lipid and glucose metabolism, and mechanical stress on the joint, indicating a notable correlation between OA prevalence and metabolic disorders [5].

Metabolic disorders, including obesity, hyperglycemia, dyslipidemia, and hypertension [6], disrupt bodily metabolic activities and have increasingly become public health issues in industrialized nations since the 20th century. The World Obesity Atlas 2023 indicates that overweight and obesity affect 38% of the global population [7]. The World Health Organization (WHO) has projected that by 2025, 21% of women and 18% of men globally will meet the clinical criteria of obesity [8]. Additionally, the Diabetes Atlas reported in 2021 that 537 million individuals globally are living with diabetes [9]. Epidemiological studies highlight a concurrent increase in metabolic disorders and OA. Specifically, patients with co-existing OA and metabolic disorders experience more severe symptoms compared to the population without metabolic disorders [10]. Furthermore, recent investigations including two cohorts reveal that gut microbial metabolites affect the progression of hand OA [11]. Thus, OA is not solely an age-associated disease, but also a “metabolic disorder.”

**Figure 1.**
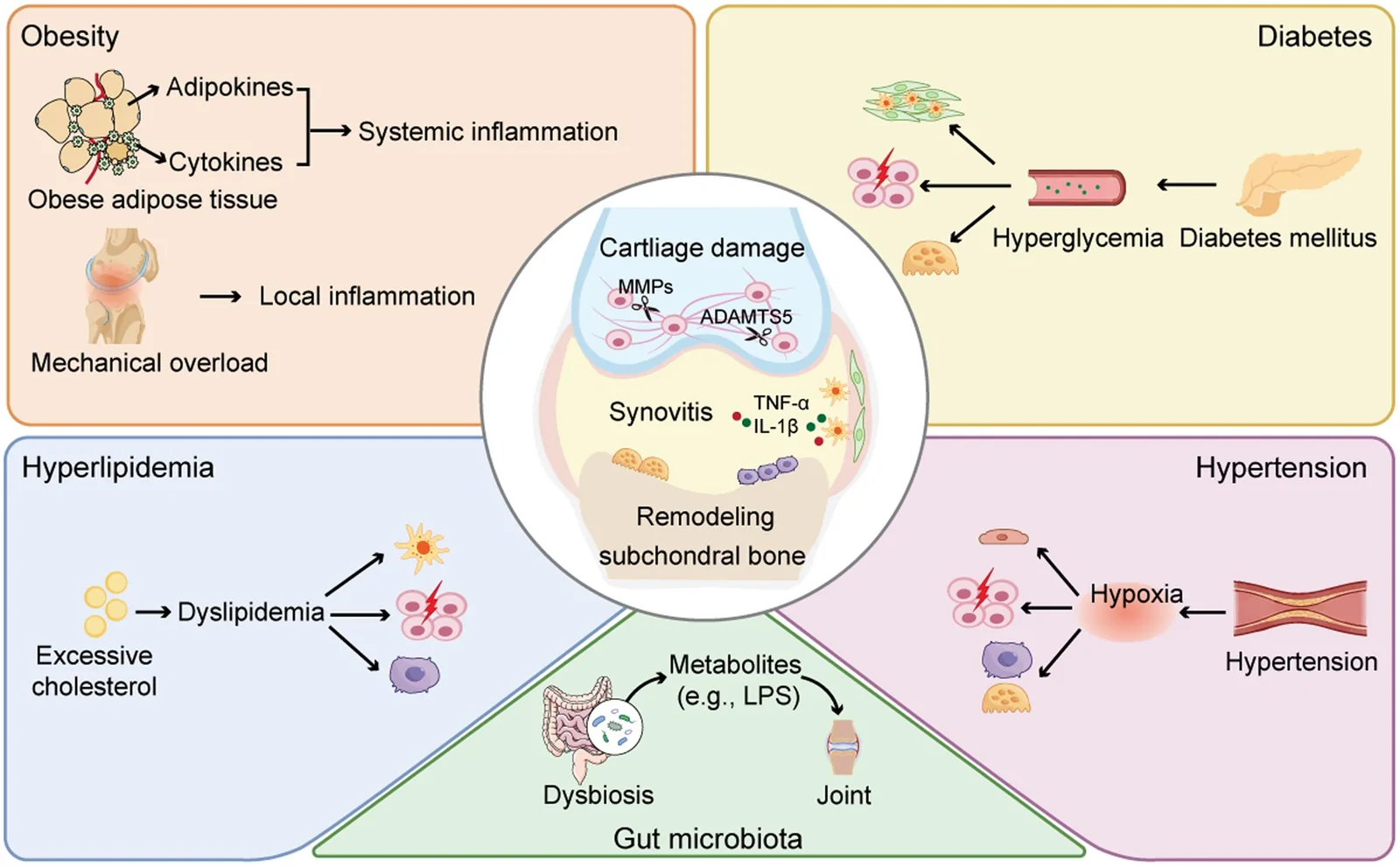
Schematic illustration of metabolic disorders-driven mechanism in osteoarthritis pathogenesis.

In this framework, multiple interrelated metabolic pathways collectively govern the initiation and progression of the OA disease (Fig. 1). Metabolic diseases, such as obesity, diabetes, dyslipidemia, hypertension, and gut microbiota dysbiosis, influence OA either independently or collaboratively through different signaling pathways. This review will discuss the epidemiological links between these prevalent metabolic disorders and OA pathogenesis, while dissecting the multi-dimensional molecular mechanisms that drive OA progression. Moreover, current clinical and preclinical therapeutic approaches targeting metabolic disease-associated OA are also summarized, which show great potential to decelerate OA progression.

## Obesity and OA

Obesity, characterized by a body mass index (BMI) of 28 kg/m^2^ or greater, and being overweight (BMI 25–28 kg/m^2^), are significant risk factors for OA [12]. Since 1986, studies from the first National Health and Nutrition Examination Survey (HANES I) confirmed the close link between obesity and knee OA (KOA) in both sexes, notably stronger in women [13]. Subsequently, a Framingham cohort study in 1988 validated this link, finding a substantial and continuous correlation between overweight or obese status in adults in their thirties and the onset of KOA around 36 years later [14]. Further analysis involving 3068 individuals aged 45 and older reinforced this relationship, indicating that the lifetime risk of experiencing symptomatic KOA increases with BMI, reaching nearly two-thirds among the obese [15]. Overall, these studies consistently highlight the elevated risk of OA in obese individuals and suggest that both abnormal mechanical loads on weight-bearing joints and non-mechanical metabolic factors are primary contributors to obesity-related OA.

### Abnormal mechanical loading in obesity-associated osteoarthritis

**Figure 2.**
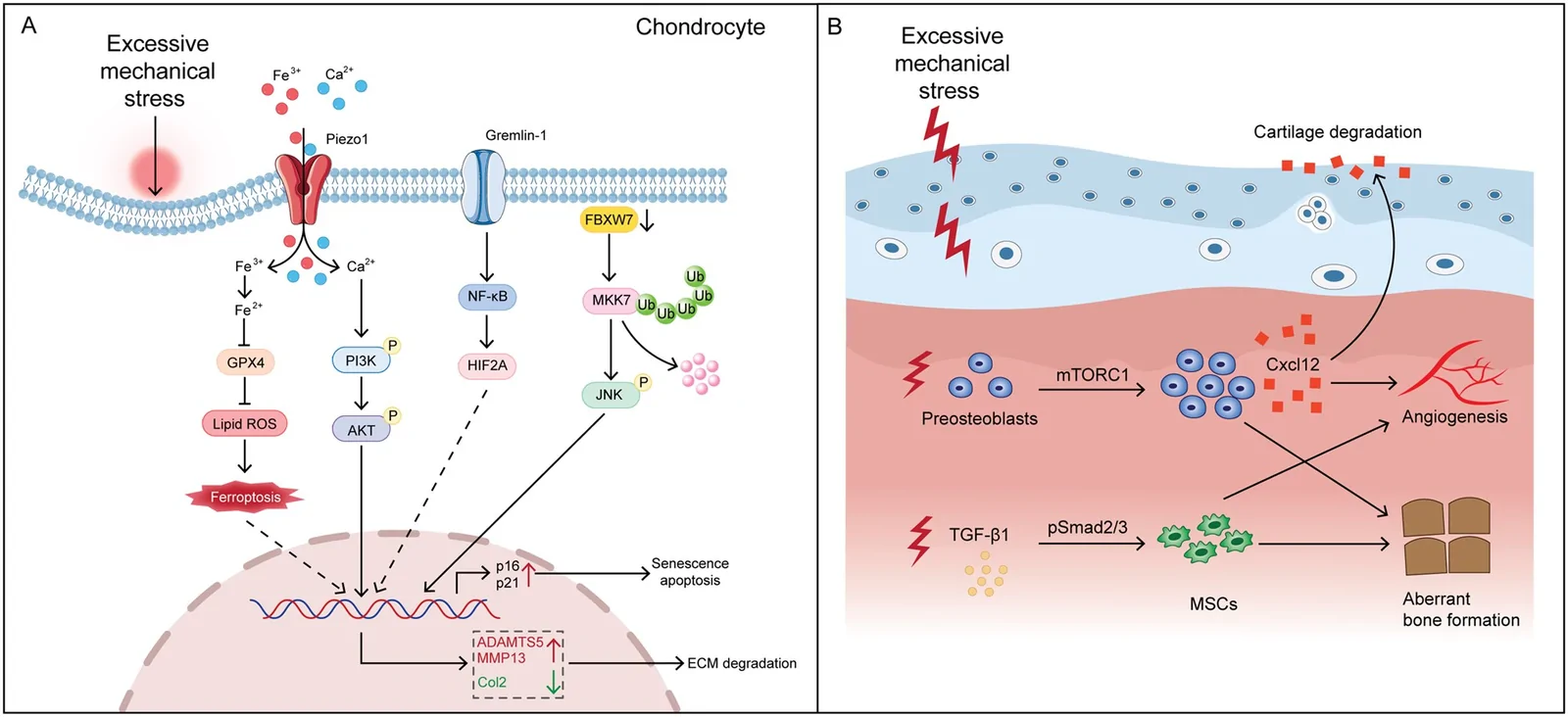
Pathophysiological mechanisms linking obesity-induced abnormal joint loading to biomechanical dysfunction in osteoarthritis. (A) Chondrocytes often express various mechanosensitive proteins. In the joints of obese individuals, excessive mechanical stress activates Piezo1, a mechanosensitive transmembrane protein in the chondrocytes. Activation of Piezo1 promotes Fe^3+^ influx into chondrocytes and induces ferroptosis by inhibiting the GPX4 pathway. It also enhances Ca^2+^ influx into chondrocytes, which decreases the expression of the anabolic biomarker Col2 and increases the expression of the catabolic enzymes MMP13 and ADAMTS5 in chondrocytes in a PI3K–AKT dependent manner. Mechanical stress upregulates Gremlin-1 expression in chondrocytes, which in turn enhances NF-κB and promotes the activation of catabolic enzymes. FBXW7, as a ubiquitin ligase, was downregulated by mechanical overloading, leading to MKK7 degradation, which subsequently activated JNK signaling, thereby inducing chondrocyte senescence and apoptosis. (B) Aside from cartilage deterioration, subchondral bone sclerosis accompanied by angiogenesis at the osteochondral junction are also characteristics of OA pathology. In response to abnormal mechanical loading, TGF-β1 is activated in the subchondral bone to increase phosphorylation of Smad2/3. This induces the formation of nestin^+^ MSC clusters, which drive abnormal bone formation and angiogenesis, thereby facilitating OA progression. Concurrently, mechanical stress also activates mTORC1 signaling in the subchondral compartment, stimulating excessive proliferation of preosteoblasts and enhancing the secretion of Cxcl12.

Proper mechanical loading is critical for maintaining joint homeostasis; however, being obese with excessive mechanical stress on weight-bearing joints can lead to degenerative lesions [16]. Chondrocytes, which are the primary mechanosensitive cells in articular cartilage, respond to high-intensity mechanical stress by activating mechanoreceptors and signaling pathways that upregulate metalloproteinase (MMP) expression, accelerating catabolic processes [17, 18]. Notably, the mechanosensitive ion channel Piezo1, a large transmembrane protein, has been implicated in OA pathogenesis [19]. Abnormal mechanical stress activates Piezo1 in chondrocytes, triggering Ca^2+^ influx that upregulates catabolic enzymes MMP13 and a disintegrin and MMP with thrombospondin motifs 5 (ADAMTS5), via the phosphatidylinositol 3-kinase/protein kinase B (PI3K/AKT) pathway [20]. Piezo1 also induces chondrocyte ferroptosis by inhibiting the glutathione peroxidase 4 (GPX4) pathway [21]. Another mechanically responsive factor, Gremlin-1, is highly expressed in the middle and deep zones of cartilage after excessive mechanical loading. Overexpression of Gremlin-1 activates nuclear factor-κB (NF-κB) signaling, which triggers the hypoxia-inducible factor (HIF)-2α–MMPs axis and further accelerates extracellular matrix (ECM) breakdown [22]. Furthermore, recent genetic evidence revealed that F-box and Trp-Asp (WD) repeat domain containing 7 (FBXW7), an ubiquitin E3 ligase, plays a vital role in mechanotransduction, is downregulated by mechanical stress, activating mitogen-activated protein kinase kinase 7 (MKK7)-c-Jun N-terminal kinase (JNK) signaling that promotes chondrocyte senescence and exacerbates OA progression [23]. In summary, excessive mechanical stress from obesity enhances OA through the activation of various mechanoreceptors that drive chondrocyte catabolism, cell death, and senescence, ultimately resulting in irreversible cartilage degradation (Fig. 2A).

In addition to its role as a mechanical scaffold for articular cartilage during joint movement, subchondral bone adapts to mechanical changes through continuous modeling and remodeling [24]. During OA development, abnormal mechanical loading induces transforming growth factor (TGF)-β1 activation in subchondral bone, which in turn promotes the migration of bone marrow mesenchymal stem cells (MSCs) and stimulates the abnormal bone formation. Research indicates that inhibiting TGF-β signaling in MSCs may mitigate cartilage degeneration in OA mouse models [25]. Additionally, activation of mammalian target of rapamycin complex 1 (mTORC1) in Osterix^+^ preosteoblasts within the subchondral bone of OA mice leads to increased proliferation of pre-osteoblast clusters, accompanied by higher secretion of C–X–C motif chemokine ligand 12 (Cxcl12), which further contributes to cartilage degradation and accelerates OA progression [26]. Collectively, these findings highlight a critical cellular crosstalk between articular chondrocytes and bone-forming cells (Fig. 2B).

Nevertheless, mechanical factors alone do not sufficiently explain the link between adiposity and the onset of OA. For instance, lifelong runners who experience greater joint loads than those attributed to obesity do not show a consistent increase in OA risk [27]. Additionally, changes in biomechanical patterns cannot account for the increased OA risk in non-weight-bearing joints, like the hand, observed in obese individuals [28]. This indicates that systemic, non-mechanical factors may play a significant role in obesity-related OA, beyond just the impact of weight on weight-bearing joints.

### Non-mechanical risk factors in obesity-associated osteoarthritis

Experimental studies demonstrated that obesity aggravates joint degeneration through systematic inflammatory mechanisms independent of mechanical stress. For instance, obese rats undergoing anterior cruciate ligament-transection (ACLT) surgery showed that comparable cartilage destruction in both obese contralateral joints and injured limbs, suggesting that obesity alone may induce OA [29]. Another study showed that a high-carbohydrate/high-fat diet led to obesity-related synovitis in rats, characterized by pro-inflammatory macrophage activation and OA-like changes [30]. These results underscore the significant role of obesity in OA development, extending beyond the effects of BMI.

### Adipose tissue-secreted adipokines contribute to OA in obesity

Individuals with obesity possess significant white adipose tissue, which is not only a storage site for energy but also functions as a powerful endocrine organ that secretes pro-inflammatory and immunomodulatory cytokines [31, 32]. Adipokines, released by white adipose tissue, have adverse systemic effects on joints and are suggested as a link between obesity and OA [33]. Currently, several adipokines, such as leptin, resistin, adiponectin, and visfatin, are hypothesized to play significant roles in OA associated with obesity.

#### Leptin

Leptin is a 16-kDa peptide encoded by the *Lep* gene and is recognized as the prototypical adipokine. It was discovered in 1994 by Friedman’s research team, who cloned the mouse *obese* (*ob*) gene and synthesized the corresponding protein, named leptin [34]. This discovery enhanced our understanding of adipose tissue’s role in obesity and prompted various studies to explore leptin’s function in OA pathogenesis. In OA-affected joints of obese patients, leptin levels in synovial fluid correlated positively with BMI, and immunohistochemical analysis indicated that leptin expression is associated with cartilage degradation severity [35]. Additional studies also demonstrated that leptin upregulates the production of MMP1 and MMP3 in human OA cartilage [36]. Similarly, leptin intra-articular injection in rat OA models leads to accelerated articular cartilage destruction, indicating a catabolic role in cartilage metabolism and OA pathology [37]. Beyond its local effects on joints, systemic leptin levels also correlated positively with BMI and were generally higher in women than in men [38]. Both cross-sectional and longitudinal studies found an independent association between systemic leptin and reduced cartilage thickness [39]. Research using leptin-deficient (*ob/ob*) and leptin receptor-deficient (*db/db*) mice indicates that leptin signaling is crucial for producing systemic inflammation and for the progression of KOA, suggesting it drives joint cartilage degradation in OA, aside from its role in obesity [40].

#### Adiponectin

Adiponectin, an adipokine secreted by adipocytes, engages with adiponectin receptor 1 (AdipoR1) and AdipoR2 receptors [41]. Studies indicate that individuals with OA have higher adiponectin levels in plasma and synovial fluid compared to healthy controls [42–46]. Additionally, elevated serum adiponectin is associated with more severe radiographic and clinical signs in KOA, though not in hand OA [36, 44, 45]. Synovial fluid adiponectin shows a stronger association with KOA severity in women, suggesting gender-specific involvement [46]. In obese patients, adiponectin production rises with cartilage lesion severity, unlike in non-obese individuals [47]. Meanwhile, treatment with adiponectin on cartilage samples from OA patients induced MMPs production and increased collagenase-cleaved type 2 collagen neoepitope expression [48]. Taken together, these results provide evidence that adiponectin may play a role and contribute to the breakdown of the cartilage matrix in OA.

#### Visfatin

Visfatin (pre-B-cell colony-enhancing factor) has been identified as another key adipokine and exerts a harmful influence in the progression of OA [49]. Clinical evidence has revealed that visfatin levels of both serum and synovial fluid were notably higher in those affected by OA than in healthy joints [50, 51]. Furthermore, in OA patients, synovial fluid visfatin levels are higher in individuals with more severe radiographic joint damage [52]. Visfatin is linked to toll-like receptor 4 (TLR4) to drive cartilage destruction by dose-dependently increasing the production of MMP3 and MMP13, ADAMTS4 and ADAMTS5 in the articular chondrocytes [53]. Notably, visfatin is expressed in chondrocytes in OA patients and contributes to cartilage destruction by disrupting ECM homeostasis [54]. Furthermore, visfatin promotes osteophyte formation by altering MSC differentiation [55].

#### Resistin

Resistin is a pro-inflammatory adipokine increasingly recognized as a mediator linking obesity and OA, primarily secreted by macrophages in humans [56]. Epidemiological and clinical studies have demonstrated that resistin levels in both serum and synovial fluid are elevated in individuals with obesity-associated OA relative to non-obese controls, with these increased levels positively correlating with adiposity and synovitis severity in OA patients [49, 57, 58]. Specifically, the levels of resistin in synovial fluid but not in serum are positively associated with symptomatic OA [59], suggesting locally elevated resistin concentrations are more detrimental to the initiation and progression of OA. Consistently, animal studies have demonstrated that rats subjected to a short-term high-fat diet exhibit increased resistin expression in chondrocytes, which in turn promotes OA progression by the release of inflammatory cytokines and enhancing the production of MMPs [60]. Accordingly, resistin has been considered as one of the key adipokines that characterizes the obesity-associated OA phenotype, and might serve as a potential biomarker for early identification of metabolic-related joint destruction.

Collectively, adipokines released by adipocytes are closely associated with BMI and OA severity, and they drive cartilage matrix breakdown, inflammation, and osteophyte formation. Recent advancements in clinical research have led to the identification of new adipokines, suggesting that targeting adipokine-related signaling pathways could be a promising strategy for the prevention and management of OA.

### Adipose tissue macrophage-mediated inflammation facilitates OA

Along with adipokines, adipose tissue also contains diverse immune cells and blood arteries [61]. It is widely accepted that obesity represents a state of low-level chronic inflammation [62]. Notably, this inflammatory response is activated early during adipose tissue expansion and persists throughout obesity, driving a sustained shift of the immune system toward a pro-inflammatory phenotype [63]. A hallmark of adipose-associated inflammation is the infiltration by various immune cells, including macrophages, neutrophils, T cells, and B cells into adipose depots [64]. Among these, macrophages are central mediators of obesity-associated chronic inflammation and play a pivotal role in OA progression. Research suggests that expanding adipocytes and surrounding pre-adipocytes secrete chemotactic signals to recruit macrophages that originate from circulating monocytes in the bloodstream [42]. For example, monocyte chemoattractant protein 1 (MCP-1), also referred to as C–C motif chemokine ligand 2 (CCL2), has been identified as a strong chemotactic factor regulating the migration and infiltration of monocytes [65]. A previous study has confirmed that macrophage accumulation was markedly increased in adipose tissue under obese conditions in both humans and mice [66]. Importantly, Obin et al. further reported that over 90% of macrophages in the white adipose tissue of obese mice and humans are concentrated around necrotic adipocytes [67]. These macrophages fuse to form multinucleate giant cells that phagocytose lipid droplets from necrotic adipocytes, marking chronic inflammation. While this infiltration clears dead adipocytes and limits tissue expansion, it also sustains local inflammation.

Typically, macrophages in adipose tissue are categorized into two subsets, pro-inflammatory M1 and anti-inflammatory M2 macrophages. M1 macrophages secrete inflammatory cytokines, including interleukin IL-1β, IL-6, and tumor necrosis factor TNF-α, while M2 macrophages release anti-inflammatory cytokines such as IL-10 and TGF-β [68, 69]. Accumulating evidence indicates that during adipose tissue expansion, macrophage populations undergo dramatic changes, with a rise in the number of infiltrating M1-polarized macrophages, which exhibit an obvious pro-inflammatory phenotype [42]. Pro-inflammatory cytokines contribute to cartilage matrix degradation and bone resorption by suppressing the synthesis of proteoglycans and type II collagen, while enhancing the production of MMPs and prostaglandins to amplify matrix breakdown [70, 71]. Furthermore, macrophages contribute to OA through TNF-α–mediated paracrine crosstalk with adipocytes, altering adiponectin and leptin synthesis to boost TNF-α and sustain local inflammation [72]. Leptin exerts pro-inflammatory effects and drives complex macrophage phenotypic regulation, whereas adiponectin exerts anti-inflammatory effects, promotes M2 polarization, and inhibits M1 activation [73–77].

Collectively, obesity-related systemic low-grade inflammation worsens OA, primarily through mediators from adipose tissue, like adipokines and pro-inflammatory cytokines released by macrophages. These substances foster a self-reinforcing inflammatory loop that accelerates OA progression in obese individuals.

### Treatment of obesity-associated OA

#### Weight loss

Obesity-associated OA is influenced by mechanical overload and persistent low-grade inflammation, necessitating a management approach that addresses weight, biomechanics, and inflammation collectively. Weight reduction is essential for treating obesity-associated OA, with a network meta-analysis revealing that bariatric surgery, a low-calorie diet with exercise, and intensive weight loss with exercise yield the most significant reductions in Western Ontario and McMaster Universities Osteoarthritis Index (WOMAC) pain scores [78]. Each 1% weight loss was correlated with 2% points reduction in WOMAC pain, function, and stiffness, whereas around a 25% weight loss was required to achieve a 50% reduction in WOMAC scores [78]. Moreover, a recent network meta-analysis showed that a combined diet with exercise was the only strategy significantly better than control for pain, and clinically important pain relief generally appears once ≥ 7% weight loss is achieved [79]. Overall, scoping and narrative reviews indicate that a body weight decrease of at least 7% through exercise results in significant pain alleviation, functional improvements, and enhanced quality of life.

#### Pharmacologic strategies in obesity-related OA

Standard analgesic treatments for OA, including nonsteroidal anti-inflammatory drugs (NSAIDs), corticosteroid injections, and hyaluronic acid, show reduced effectiveness and greater adverse effects in obese patients, necessitating incorporation into a comprehensive weight management strategy [80]. A large-scale randomized controlled trial (RCT) with 407 individuals suffering from obesity and KOA demonstrated that weekly subcutaneous semaglutide at 2.4 mg led to a weight reduction of 13.7%, compared to 3.2% in the placebo group. The treatment also resulted in a significant decrease in WOMAC pain scores (41.7 vs. 27.5) and improved physical function over 68 weeks, while maintaining similar serious adverse event rates as the placebo [81]. Another RCT of women with KOA and obesity, adding orlistat to diet and exercise resulted in approximately 10% weight loss and nearly 50% reductions in WOMAC pain, concomitant with decreased levels of leptin and IL-6, indicating that orlistat in conjunction with exercise possesses significant anti-inflammatory properties [82]. These findings suggest obesity-targeted pharmacotherapies (e.g. orlistat, glucagon-like peptide-1 (GLP-1) receptor agonists such as semaglutide) alleviate OA pain primarily in relation to weight reduction and decrease inflammation.

Interestingly, our latest study identified a novel mechanism by which semaglutide mitigates KOA independently of weight loss [83]. In pair-feeding obese OA mice with equivalent weight reduction, only semaglutide treatment exerted significant chondroprotection. It shifted chondrocyte metabolism from glycolysis to oxidative phosphorylation via the GLP-1R–AMP-activated protein kinase (AMPK) pathway, facilitating cartilage restoration [83]. To definitively determine if weight-loss–associated improvement of OA function is due to reduced joint stress or restoration of the metabolic disorder, more clinical studies need to be conducted.

## Diabetes and OA

Similar to obesity, diabetes is closely associated with OA. Diabetes is characterized by chronic hyperglycemia and has grown increasingly prevalent worldwide [84, 85]. A 20-year longitudinal cohort study involving 927 participants suggested that type 2 diabetes mellitus (T2DM) predicted both joint failure and hip or knee arthroplasty independent of these confounders [86]. Similarly, another study reported that after adjusting for these two factors (age and BMI), individuals with T2DM had higher odds of receiving a clinical diagnosis of knee or hand OA [87]. Furthermore, the recent two meta-analyses consistently indicated that patients with T2DM face a moderately increased risk of developing OA compared to those without diabetes [88, 89]. A cohort study in the United States with 179 participants found that individuals with both KOA and diabetes reported significantly greater knee pain, either unilateral or bilateral, even after controlling for BMI and analgesic treatments [90]. Furthermore, inadequate glycemic management has been associated with greater pain severity in patients suffering from both OA and T2DM [91]. These findings suggest that diabetes is linked to elevated OA risk, more severe joint damage, and worse pain, independently of age and BMI.

To further explore the potential molecular mechanisms several classic animal models of diabetes, including streptozotocin (STZ)-induced diabetic models, *db/db* mice (spontaneous) models, and high-fat diet combined with STZ models have been widely utilized [92]. Evidence indicates that diabetes contributes to the pathogenesis of OA through multiple pathways, notably synovial inflammation, cartilage degradation, and subchondral bone remodeling [93].

### Diabetes-induced synovitis

Both OA and diabetes are chronic inflammatory diseases linked to increased levels of IL-6 in hyperglycemic patients, leading to more severe synovitis compared to normoglycemic individuals [94]. Experimental models involving high-fat diet and STZ treatment in rodents demonstrate worsened synovial inflammation marked by macrophage infiltration and elevated intracellular adhesion molecule 1 (ICAM-1) expression [95]. Synovial angiogenesis further enhances local inflammation by facilitating immune cell recruitment [96], while high glucose conditions induce pro-angiogenic factors in synovial fibroblasts through oxidative stress, worsening synovial inflammation [97]. Consistent with these findings, results from studies involving diabetic OA patients show more severe synovitis and higher macrophage abundance in the synovium compared with non-diabetic OA counterparts [98]. Fibroblast-like synoviocytes (FLSs) from OA patients with T2DM exhibit insulin resistance at the molecular level, evidenced by impaired insulin receptor and AKT phosphorylation [98]. A recent study identified a key metabolic pathway in which increased glucose transporter 1 (GLUT1)-dependent glycolysis, driven by yes-associated protein 1 (YAP1)/thioredoxin-interacting protein (TXNIP) signaling axis in FLSs, promotes M1-polarization of synovial macrophages in diabetic OA patients and *db/db* diabetic OA mice [99]. These findings suggest that FLSs contribute to synovitis and macrophage metabolism in diabetic OA, while inflammatory macrophages enhance FLS proliferation, creating a self-perpetuating inflammatory cycle.

### Diabetes-induced abnormal cartilage metabolism

Articular cartilage is an avascular and aneural connective tissue composed of a dense ECM, rich in type II collagen, proteoglycans and water, maintained by resident chondrocytes. Its primary function is to minimize friction and distribute mechanical loads across the joint. In diabetes, excessive accumulation of advanced glycation end products (AGEs) within cartilage promotes oxidative stress in chondrocytes and accelerates cartilage loss [100]. AGEs bind to TLR4 and the receptor for advanced glycation end products (RAGE) on chondrocytes, triggering activation of the NF-κB signaling pathway and subsequent upregulation of MMPs and pro-inflammatory cytokines [101]. Chondrocytes regulate glucose homeostasis by adjusting GLUT1 synthesis and degradation according to extracellular glucose levels [102]. Consistent with these observations, diabetic mice (*db*/*db* mice) following experimental OA induction exhibit more severe cartilage damage compared with lean controls [97]. Results of *in vitro* studies further indicate that high glucose levels can decrease the transport of dehydroascorbic acid into chondrocytes, reducing the synthesis of type II collagen [103], while decreasing cartilage anabolism and enhancing MMPs expression [104]. Conversely, low glucose concentrations promote the expression of aggrecan in mouse chondrocytes [105], suggesting that moderate glucose levels may prevent OA progression, while high glucose accelerates cartilage degeneration and OA progression.

### Diabetes-induced subchondral bone destruction

A small-scale study observed significant bone loss at the subchondral plate in KOA patients who had both hypertension and T2DM [106]. In animal models, diabetes has also been shown to worsen subchondral bone health and accelerate OA progression. In STZ-induced diabetic mice subjected to the destabilization of the medial meniscus (DMM) surgery, diabetes deteriorated the subchondral bone microarchitecture, marked by a significant reduction in bone mass and rapid breakdown of trabecular structure [107]. Furthermore, a comparison of KOA patients revealed that those with T2DM had abnormal remodeling in the subchondral bone of the tibial plateaus, marked by reduced bone formation and increased osteoclast activity [108]. Previous studies indicate that during OA progression, osteoclasts in subchondral bone were activated to release netrin-1, which promotes sensory nerve axon growth linked to OA-related pain behaviors [109, 110]. This may account for the more severe joint pain often reported by diabetic patients. However, the relationship between diabetes and OA is not clearly understood, necessitating further cohort studies and animal research to explore the molecular mechanisms involved.

**Figure 3.**
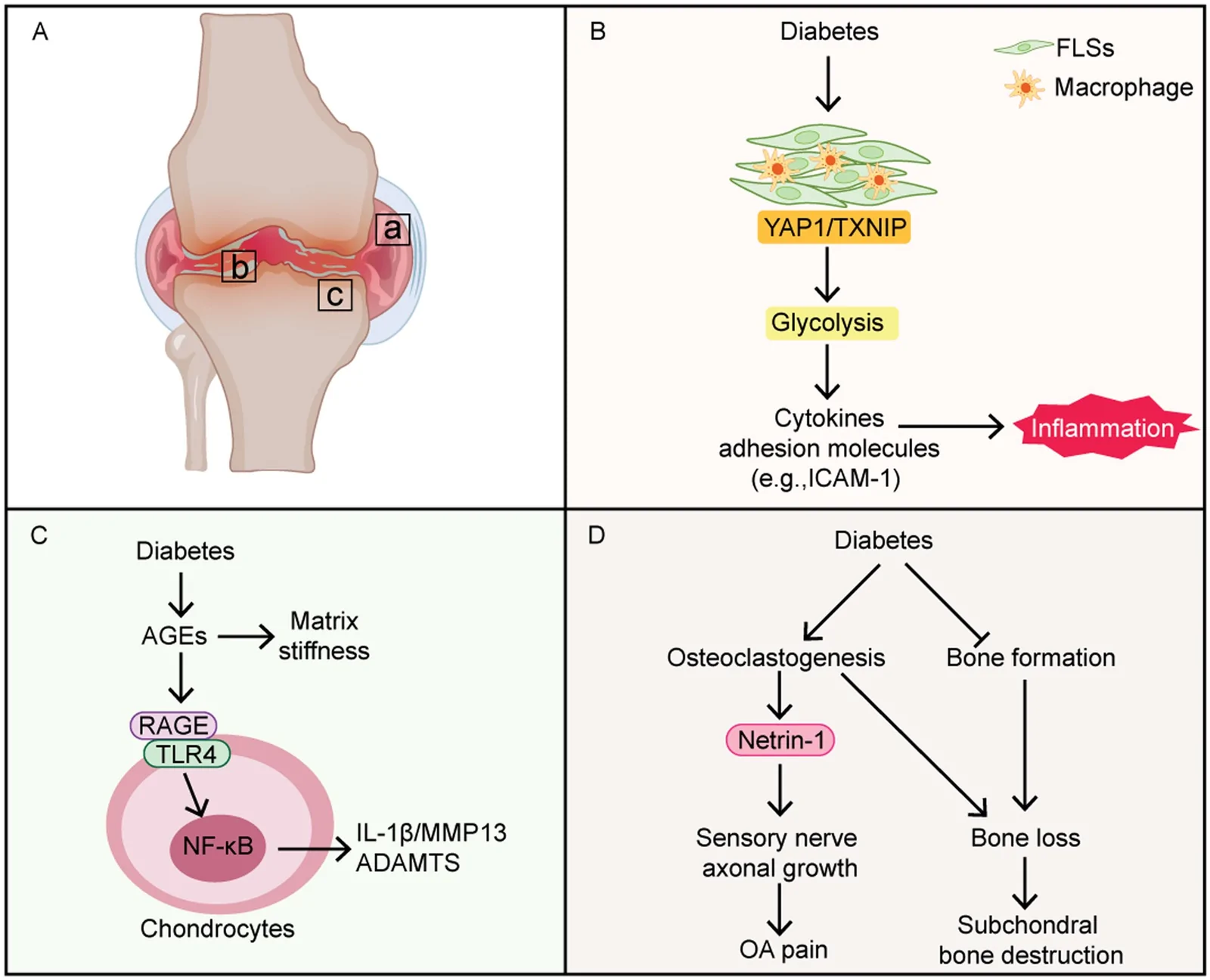
The potential mechanistic role of diabetes in the pathogenesis of osteoarthritis (OA). (A) Schematic diagram showing the three major joint compartments targeted by pathological changes in diabetic OA: (a) synovial tissue, where FLSs and macrophages mediate synovial inflammation; (b) articular cartilage, which undergoes progressive degradation; and (c) subchondral bone, which is prone to remodeling abnormalities. (B) During diabetes-associated OA, joint-resident FLSs and macrophage interaction creates a cytokine network that activates an inflammatory response in the synovium. Increased GLUT1-dependent glycolysis controlled by YAP1/TXNIP signaling axis in FLSs drives M1polarization of synovial macrophages in diabetic OA, leading to exacerbated synovitis. (C) Diabetes-induced AGE in chondrocytes binds to TLR4 and RAGE, increasing the production of downstream proteins such as MMPs and pro-inflammatory factors through NF-κB signaling, resulting in accelerated cartilage breakdown. (D) The subchondral bone of diabetes OA shows a bone loss phenotype, characterized by a decrease in bone formation and an increase in osteoclastogenesis. Additionally, activated osteoclasts cause sensory nerve sprouting in the subchondral bone, which plays a critical role in OA pain.

Diabetes exacerbates OA by promoting synovitis through increased macrophage infiltration, elevated pro-inflammatory cytokines, and impaired insulin signaling in FLSs. In cartilage, diabetes-induced AGE accumulation activates NF-κB, increasing MMPs, while high glucose disrupts chondrocyte metabolism, impairing collagen synthesis and accelerating degeneration. Moreover, diabetic OA is linked to subchondral bone destruction, characterized by osteoclast overactivity, reduced bone formation, and worsened pain due to nerve growth. These findings suggest diabetes amplifies OA severity through inflammatory, cartilage matrix degradation, and subchondral bone destruction (Fig. 3).

### Antidiabetic agents for diabetes-related OA

In diabetes-related OA, medications like paracetamol (acetaminophen), NSAIDs, and intra-articular corticosteroids can relieve pain but raise safety concerns due to comorbidities and drug interactions, requiring careful risk-benefit evaluation and monitoring. Conversely, oral glucosamine and intra-articular hyaluronic acid are safer options for patients with T2DM and OA [92, 111].

Several antidiabetic agents may influence OA progression through shared inflammatory and metabolic pathways. Metformin has been shown to activate AMPK, reducing synovitis and cartilage degradation, thereby limiting OA progression [112]. When combined with cyclooxygenase-2 (COX-2) inhibitors, metformin can further reduce OA progression and joint replacement rates in patients with both OA and T2DM [113]. GLP-1 receptor-based therapies not only improve metabolic profiles but also demonstrate anti-inflammatory and chondroprotective effects, potentially aiding in cartilage repair [111]. In a large T2DM cohort, sodium-glucose cotransporter 2 (SGLT2) inhibitors were associated with lower risks of new-onset OA and joint replacement compared with GLP-1 receptor agonists [114]. Beyond glycemic control, insulin may modulate inflammatory pathways relevant to joint health, though supporting data remain in early stages [115]. Additionally, the use of alpha-lipoic acid (ALA) alongside standard therapy for OA and T2DM showed greater improvements in pain, likely due to its antioxidant and metabolic properties [116]. Recently, mesenchymal stromal/stem cell-derived exosomes (MSC-Exos) show potential for repairing cartilage and managing blood sugar levels. However, further research on safety, dosing, and long-term efficacy is essential [117]. Broader stem cell based and tissue-engineering strategies remain under investigation without established clinical application in diabetes-related OA.

## Hyperlipidemia and OA

Hyperlipidemia, characterized by elevated plasma levels of total cholesterol, triglycerides, and low-density lipoprotein (LDL) cholesterol, alongside reduced high-density lipoprotein (HDL) cholesterol [118, 119], is linked to cardiovascular diseases like atherosclerosis [120]. Accumulating studies have established a close association between hyperlipidemia and OA [121, 122]. Patients with radiographic KOA had increased cholesterol, triglycerides and LDL cholesterol [123]. Moreover, elevated total cholesterol and decreased HDL cholesterol levels correlate closely with increased severity of knee pain in OA [124]. A Mendelian randomization study involving 376,435 adults from the UK confirmed the causal relationship between elevated LDL cholesterol levels and increased OA risk [122]. Recent studies from South Korea and Russia found that individuals with hypercholesterolemia have a higher prevalence of KOA and report more severe joint pain compared to those with normal cholesterol levels [124, 125].

### Cartilage damage by disturbed cholesterol metabolism

Cholesterol, a ubiquitous component of membranes in most mammalian cells including chondrocytes, plays an indispensable role in regulating the stability, fluidity, and permeability of lipid bilayers [126]. Cholesterol metabolism is tightly regulated by biosynthesis, influx of lipoprotein cholesterol into cells, and efflux from cells [127, 129]. The maturation of sterol regulatory element-binding protein 2 (SREBP-2) in the endoplasmic reticulum activates cholesterol influx. This results in the transport of SREBP-2 into the nucleus, where it initiates the transcription of genes involved in cholesterol biosynthesis and uptake, particularly 3-hydroxy-3-methylglutaryl coenzyme A (HMG-CoA) reductase and the LDL receptor (LDLr) [128, 129]. On the other hand, when cholesterol is accumulated in cells, the liver X receptors α and β (LXRα and LXRβ) trigger reverse cholesterol transport to remove deposited cholesterol from cells [130]. LXRs promote cholesterol efflux by directly upregulating genes such as the ATP-binding cassette (ABC) transporter A1 (ABCA1) and G1 (ABCG1). This activity facilitates the transfer of excess cellular cholesterol to apolipoprotein A1 (ApoA-1) and HDL particles, a key mechanism for preventing hypercholesterolemia [131, 132]. The primary HDL component, ApoA-1, is crucial for cholesterol efflux and lipoprotein clearance.

High cholesterol has negative impact on articular cartilage by disrupting lipid homeostasis through a specific molecular pathway. This process is associated with increased expression of key modulators of cholesterol uptake, including retinoic acid-related orphan receptor alpha (RORα), cholesterol 25-hydroxylase (CH25H), lectin-type oxidized low density lipoprotein receptor 1 (LOX-1), and 25-hydroxycholesterol 7α-hydroxylase (CYP7B1) [133]. It has been reported that excess cholesterol accumulation induced by disturbed cholesterol metabolism aggravates OA cartilage degeneration by ECM degradation [134]. The receptor LOX-1, which binds oxidized LDL, is significantly overexpressed in the chondrocytes of both human OA patients and experimental animal models [135]. Genetic deletion of LOX-1 inhibits OA development by preventing chondrocyte hypertrophy and apoptosis [136, 137]. In line with this finding, research by Choi et al. found that a cholesterol-rich diet exacerbates joint damage in DMM-operated mice and promotes cholesterol accumulation in chondrocytes. The overexpression of LOX-1 in chondrocytes mediated lipid uptake drives the activation of the CH25H–CYP7B1–RORα axis, which in turn transcriptionally upregulates catabolic enzymes such as MMP3 and MMP13 [138]. Furthermore, recent findings indicate that LDL receptor-related protein 3 (LRP3) is significantly diminished in the cartilage of mice subjected to a high-cholesterol diet, subsequently leading to the upregulation of syndecan-4 (SDC4) through the activation of the Ras/Raf/MEK/ERK signaling pathway, resulting in the degradation of ECM [139]. Thus, LRP3 positively regulates chondrocyte ECM metabolism in OA pathogenesis, with syndecan-4 identified as a key downstream molecular target.

Furthermore, accumulation of intracellular lipids in OA chondrocytes inhibits the expression of cholesterol efflux-related genes, such as ABCA1, ApoA1, LXRα, and LXRβ. Research on human OA cartilage samples has shown significantly reduced expression of these genes. Conversely, treatment with TO-901317, an LXR agonist, increases the expression of these genes and decreases lipid accumulation in chondrocytes [140, 141]. Articular chondrocytes from OA patients showed decreased LXRα and LXRβ expression after treatment with IL-1β and TNF-α, resulting in ECM degradation. However, LXR agonists mitigated the catabolic effects of these inflammatory cytokines [29]. These findings indicate that the activation of LXR signaling to facilitate cholesterol efflux in chondrocytes may contribute to the delay of OA progression.

### Synovitis and ectopic bone formation by disturbed cholesterol metabolism

LDL particles function as the primary carriers for transporting cholesterol from the liver to peripheral tissues, including the joints. Within an inflammatory joint environment, these LDL particles are susceptible to oxidation, forming oxidized LDL (oxLDL) [142]. The oxLDL level was found to increase in the synovium of individuals with OA [143]. In a series of mechanistic studies, in 2013, de Munter et al. found that serum LDL cholesterol accumulation caused by LDL receptor deficiency or a cholesterol-rich diet with collagenase-induced OA mice results in ectopic bone formation within joint ligaments [144]. This process was mediated by synovial macrophage uptake of oxLDL, which predominantly activated TGF-β signaling [144]. Apolipoprotein E (ApoE) is a crucial plasma lipoprotein that transports triglycerides and cholesterol. In 2016, research showed that a cholesterol-rich diet in *Apoe^-/-^* mice, alongside a specific experimental OA model, resulted in early ectopic bone formation and synovial thickening [145]. However, high cholesterol diets were not linked to cartilage destruction in two animal models, consistent with Villalvilla et al.’s findings that high-fat diets did not alter rabbit cartilage in an OA model [146]. Another study indicated that high-fat diet-induced hypercholesterolemia led to synovial inflammation. [147]. Thus, a high-fat diet did not affect cartilage histology but induced synovial inflammation, indicating synovitis as a key pathogenic driver. Previous studies underscore the link between inflammation and cholesterol homeostasis through transporter ABCA1 [148], which facilitates cholesterol transport to apoA-1 for HDL synthesis. Mechanistic studies showed that the interaction of apoA-1 with ABCA1 activates cholesterol efflux and STAT3 branch pathways to synergistically suppress inflammation in macrophages [149]. Consequently, agonists aimed at increasing ABCA1 expression in macrophages may help alleviate inflammation associated with OA.

**Figure 4.**
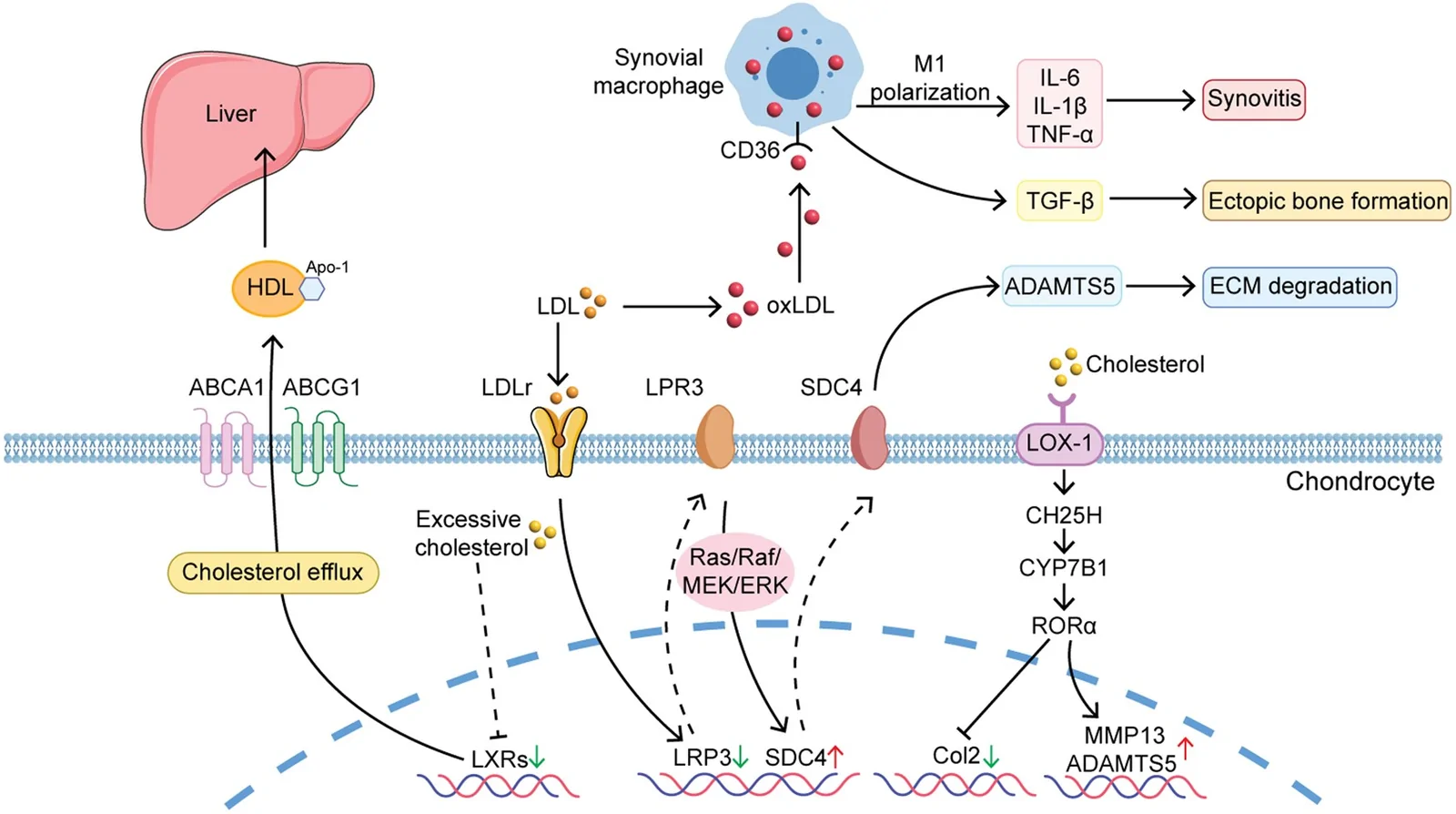
Molecular mechanisms underlying osteoarthritis pathogenesis induced by disturbed cholesterol metabolism. Serum LDL transports cholesterol to joint tissues via LDLr/LRP3 receptors, where inflammation promotes its oxidation to oxLDL. Synovial macrophages internalize oxLDL via CD36/LOX-1 receptors, which polarizes macrophages toward a pro-inflammatory phenotype, thus triggering IL-1β and TNF-α release. Meanwhile, this process activates TGF-β-mediated ectopic bone formation. Regarding cholesterol metabolism in chondrocytes, excessive lipid uptake mediated by LOX-1 drives activation of the CH25H–CYP7B1–RORα axis, which suppresses Col2 expression while upregulating catabolic enzymes MMP3, and MMP13. LDL-induced downregulation of LDL LRP3 upregulates SDC4 expression by activating the Ras/Raf/MEK/ERK signaling pathway, thereby disrupting ECM homeostasis. Concurrently, excess cholesterol impaired the cholesterol efflux pathway via downregulation of LXRs, thus reducing ABCA1/ABCG1 expression and promoting lipid accumulation.

All the above data strongly implicate that aberrant cholesterol metabolism acts as a catabolic driver of cartilage damage, accompanied by the stimulation of local pro-inflammatory mediators, worsening OA outcomes (Fig. 4). However, further studies are warranted to clarify the exact processes connecting cholesterol metabolism with OA development.

### Lipid-lowering medications for hyperlipidemia-related OA

Lipid-lowering pharmaceuticals are treatments aimed at reducing plasma triglycerides or cholesterol levels. Key options include statins, HMG-CoA reductase inhibitors, the protein convertase subtilisin-kexin type 9 (PCSK9) inhibitors, and fibrates as peroxisome proliferator receptor alpha (PPARα) activators [150]. These medications represent an innovative therapeutic approach for metabolic OA by targeting specific lipid metabolic disorders.

Statins are primarily known for lowering serum cholesterol levels by inhibiting its biosynthesis [151, 152]. Interestingly, they also show potential therapeutic effects in OA [153]. Atorvastatin attenuates monosodium iodoacetate (MIA)-induced OA pain and protects cartilage breakdown via inhibition of oxidative stress [154]. Mevastatin reduces cartilage degradation and synovial inflammation in the rabbit OA model [155]. Furthermore, statins like pravastatin, simvastatin, and fluvastatin demonstrate chondroprotective effects in human chondrocytes by decreasing MMPs enzyme activity [156, 157]. Given their multifaceted targeting of OA mechanisms, statins are proposed as potential disease-modifying drugs for OA.

Fibrates, including fenofibrate, bezafibrate, and gemfibrozil, act as PPARα agonists and are commonly used to reduce serum triglyceride levels. Among these, fenofibrate is the only fibrate currently associated with OA. Studies suggest that PPARα activation promotes fatty acid uptake and metabolism while also conferring anti-inflammatory effects [158]. In patients with hand OA, a 12-week course of fenofibrate led to improvements in pain, hand function, systemic inflammation, and lipid profiles [159]. Importantly, local administration of PPARα agonists into the infrapatellar fat pad of OA patients can effectively reduce cytokine production (e.g., IL-6, IL-8, MCP1, and IL-4) stimulated by IL-1β [153]. However, fenofibrate exerts no inhibitory impact on cartilage degradation in the STR/Ort spontaneous OA mouse model [160]. Thus, the use of fibrates in the treatment of OA still requires more in-depth studies.

Ezetimibe is a cholesterol absorption inhibitor that reduces intestinal cholesterol uptake by blocking Niemann-Pick C1-like 1 (NPC1L1) protein. It has been shown to mitigate IL-1β-induced inflammation and ECM degradation in mouse chondrocytes, indicating possible chondroprotective effects [161]. Further research is necessary to assess its effectiveness in treating OA.

PCSK9 inhibitors effectively lower cholesterol by preserving LDL receptor function and are typically combined with other lipid-lowering agents. However, a study in dyslipidemic mice showed that combined atorvastatin and PCSK9 inhibitor therapy failed to prevent cartilage degradation [162], suggesting their role in metabolic OA remains uncertain despite their efficacy in dyslipidemia.

## Hypertension and OA

Recent research highlights a significant association between hypertension and OA, with more than 50% of OA patients experiencing comorbid hypertension [163, 164]. One study indicated that 67.5% of OA patients were hypertensive, surpassing rates in non-OA participants (41.6%) and the general population (48.0%) [165]. Additionally, the severity of hypertension correlates with worsened OA pain [166]. A further study established that pulse pressure is a key factor influencing knee pain severity, positively correlating with WOMAC scores [167]. In animal experiments, hypertensive rats with OA had a higher trabecular bone area ratio than male normotensive rats with OA, indicating greater bone marrow resorption or development of subchondral bone sclerosis [168]. Hypertension and OA comorbidity lead to more severe pathological damage of subchondral bone. In addition, this remodeling may be related to the vascular etiology of subchondral bone [169]. Increased intraosseous pressure and thinning of subchondral microvessels lead to decreased subchondral blood perfusion, decreased nutrient and oxygen supply to cartilage, and ultimately cartilage degeneration [169]. The mechanism of interaction between hypertension and OA is unclear, but it has been suggested that the effect of hypertension on bone joints is due to subchondral ischemia [170]. At cellular and molecular levels, hypertension and OA share common molecular pathways, including the renin–angiotensin system, endothelin system and Wnt/β-catenin signaling pathway [171–173]. Taken together, these pathways are upregulated in hypertension, causing chondrocyte hypertrophy and an inflammatory response, leading to joint catabolism and ultimately to OA progression.

### Impaired cartilage nutrition in hypertension-induced OA

The nutrient supply of articular cartilage is complex due to its lack of blood vessels and nerve endings. It relies on the synovium and subchondral bone for nourishment. A cavity filled with synovial fluid exists between the cartilage and synovium, serving as a medium for nutrient exchange. Synovial fluid is produced by synovial cells, allowing nutrients and oxygen to diffuse through capillaries to the cartilage, where they are absorbed by chondrocytes [174, 175]. There is a thin layer of calcified cartilage between articular cartilage and subchondral bone [176, 177]. Small molecules such as glucose and nitric oxide can penetrate articular cartilage through calcified cartilage [178]. Hypertension-induced blood perfusion abnormalities can lead to bone and cartilage dystrophy, which in turn contributes to cartilage damage [179]. The consequence of hypoxia is that chondrocytes upregulate GLUT1 expression, which accelerates glucose uptake [180]. Glycolysis often occurs in this anaerobic environment and produces acidic lactate, which in turn acidifies the cartilage environment and affects the synthesis of matrix molecules [181]. Hypertension leads to increased intraosseous pressure, which in OA leads to accelerated transport of molecules from bone to cartilage, promoting cartilage damage [182].

The renin-angiotensin system (RAS) plays a central role in blood pressure. Local RAS expression has been detected in chondrocytes of both human and mice, and this local RAS is particularly important for the process of chondrocyte hypertrophy [183]. Furthermore, elevated concentrations of vascular endothelial growth factor (VEGF) and MMP13 have been shown to promote the expression of angiotensin-converting enzyme (ACE). These findings suggest that the RAS may be involved in regulating angiogenesis (new blood vessel formation) in the synovium and cartilage of OA joints [184].

### Synovial edema and inflammation in hypertension-induced OA

OA synovitis is characterized by significantly increased microvessel density and endothelial cell proliferation. Increased synovial angiogenesis depends on VEGF acting on endothelial cells and on VEGF receptor 2 (VEGFR2), which is highly expressed in endothelial cells, to promote synovial angiogenesis [185]. Synovial fluid hypoxia is negatively correlated with synovial fluid blood flow but positively correlated with cartilage damage [186]. Animal studies have demonstrated that hypertension can cause increased expression of several proteins in rat aortic endothelial cells. Aquaporin 1 is one of them. Interestingly, in synovitis, excessive expression of Aquaporin 1 contributes to the formation of joint swelling and synovial edema in OA [187]. Hypertension also exacerbates synovitis by promoting endothelial Weibel-Palade body (WPB) exocytosis, which releases inflammatory factors including IL-18 and endothelin-1 (ET1). Hypertensive stretch and hypoxia trigger WPB degranulation, while ET1 forms a positive feedback loop that further amplifies WPB release and sustains inflammation [188, 189]. Excess ET1 stimulates the production of pro-inflammatory factors and triggers the catabolism of articular cartilage and synovial thickening [190]. Hypertension affects the synovium, leading to notable swelling and inflammation. It uniquely increases microvessel density and promotes endothelial cell proliferation, enhancing synovial angiogenesis, primarily through the action of VEGF on VEGFR2, which is abundantly expressed in synovial cells [183]. Additionally, hypertension leads to the overexpression of Aquaporin 1 in synovial endothelial cells, contributing to joint swelling and synovial edema.

### Subchondral bone remodeling dysregulation in hypertension-induced OA

The blood supply of subchondral bone is rich and cancellous bone contains abundant capillaries, providing a large number of nutrients [191]. Hypertension can decrease blood flow to OA joint tissues and impair subchondral bone perfusion, causing fluid flow alterations and increased intraosseous pressure. These changes induce osteocyte apoptosis and osteoclast-mediated resorption, leading to osteonecrosis [192]. Hypertension-driven hypoxia could activate bone resorption factors such as receptor activator of nuclear factor-κB ligand (RANKL), to promote osteoclastogenesis. Meanwhile, osteoclast-mediated bone resorption releases active TGF-β, which stimulates the recruitment of MSCs that further differentiate into osteoblasts, thus contributing to subchondral bone sclerosis in OA [25]. Notably, hypertension-induced hypoxia can directly stimulate the differentiation of MSCs to osteoblasts [193, 194]. Increased IL-6 has also been found in hypertensive patients, and IL-6 interacts with macrophage colony-stimulating factor (M-CSF), stimulating osteoclast formation and exacerbating osteonecrosis in OA [195].

**Figure 5.**
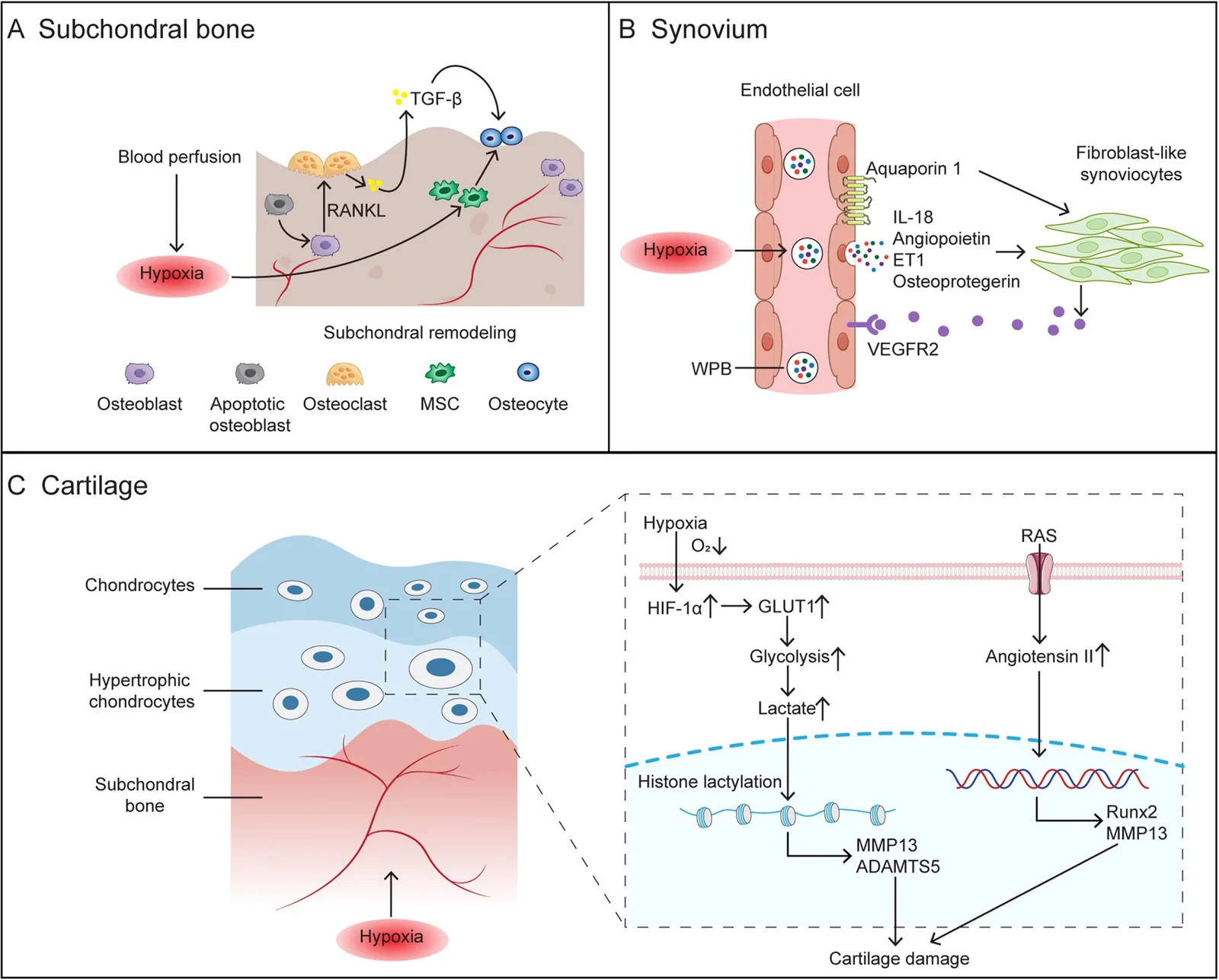
The molecular mechanism of hypertension-associated osteoarthritis in the joints. (A) Under hypoxic conditions, blood perfusion stimulates the activation of the RANKL signaling pathway, promoting the generation and activation of osteoclasts, while TGF-β facilitates the production of osteoblasts. These processes collectively participate in the remodeling of subchondral bone. (B) Endothelial cells and synovial cells (WPB) express various factors such as Aquaporin 1, IL-18, Angiopoietin, ET1, Osteoprotegerin, and VEGFR2, which promote the generation and activation of FLSs. (C) Chondrocytes and hypertrophic chondrocytes are stimulated by hypoxia, leading to increased expression of HIF-1α, which promotes the expression of GLUT1, enhancing glycolysis and lactate production. Lactate further activates MMP13 and ADAMTS5, causing cartilage damage. Additionally, hypoxia stimulates the RAS signaling pathway to promote the expression of Runx2 and MMP13, further exacerbating cartilage damage.

In summary, hypertension exacerbates OA severity and progression by enhancing subchondral bone remodeling and causing synovial swelling and inflammation, resulting in increased cartilage damage, joint pain, and functional impairment (Fig. 5). However, the specific mechanisms linking blood pressure to OA pain are not well understood, highlighting the need for further research to identify these mechanisms and create effective interventions for hypertensive patients susceptible to OA pain.

### Pharmacologic treatment in hypertension-induced OA

#### ACE and angiotensin II receptor (AR) inhibitors

These agents target the RAS, a key pathway involved in both hypertension and OA pathogenesis [196]. RAS activation promotes vascular stiffness, increases intra-articular pressure, and stimulates chondrocyte catabolism via upregulation of MMPs [196]. ACEIs (e.g., lisinopril, enalapril) and angiotensin II receptor blockers (ARBs) (e.g., losartan, valsartan) not only lower blood pressure but also inhibit MMP activity, reduce synovial inflammation, and protect cartilage from degradation [184].

#### Calcium channel blockers

Calcium channel blockers (CCBs) (e.g., amlodipine and nifedipine) lower blood pressure by blocking calcium influx into vascular smooth muscle cells, leading to vasodilation [197]. Additionally, CCBs inhibit chondrocyte apoptosis and reduce subchondral bone sclerosis, a hallmark of OA progression [198]. They are particularly suitable for hypertensive OA patients with comorbidities such as coronary artery disease.

#### Beta-blockers

While beta-blockers (e.g., metoprolol and atenolol) are effective for hypertension control, their use in OA patients requires caution [199]. Non-selective beta-blockers may reduce blood flow to synovial tissues and exacerbate joint pain in some individuals [200]. However, cardioselective beta-blockers have a more favorable safety profile and can be used in patients with concurrent cardiovascular disease and OA.

#### Acetaminophen

As a first-line analgesic for OA, acetaminophen has minimal effects on blood pressure and is suitable for hypertensive patients when used at recommended doses [201]. It relieves mild-to-moderate joint pain without anti-inflammatory properties.

#### Non-steroidal anti-inflammatory drugs

NSAIDs exert analgesic and anti-inflammatory effects by inhibiting COX enzymes, but non-selective NSAIDs can cause fluid retention, elevate blood pressure, and impair renal function [202]. Selective COX-2 inhibitors have a lower risk of cardiovascular and renal adverse effects, making them a preferred choice for moderate-to-severe OA pain in patients with controlled hypertension [203].

## Gut microbiota dysbiosis

In humans, the gut is home to trillions of microorganisms that exert a profound influence on host metabolism, immune defense, and nutritional status [204, 205]. Generally, the microbial community is highly dynamic and can be affected by several factors, including age, lifestyle, nutrition, hormone cycles, drugs, and sickness [206–208]. Disruption of the community of microbiota, called gut microbial dysbiosis, can impair gut barrier function, thereby allowing the invasion of external factors or endogenous pathobionts that affect remote organs like joints [209, 210].

### Gut microbiota

Various gut microbiomes have been found in different tissues of joints in both humans and mice with OA using 16 S rRNA microbiome sequencing. A significant revelation was the detection of *Porphyromonas* and *Bacteroides* DNA across 58 human OA synovium or synovial fluid samples [211]. Also, bacteria were observed within the calcified cartilage zone or articular–epiphysial cartilage complex, not the articular cartilage [212]. The absence of bacteria in articular cartilage may be explained by its unique avascular structure and dense ECM, which lacks blood vessels, nerves, and lymphatics, depending on nutrient diffusion from the synovial fluid and subchondral bone marrow [213]. Critically, the average pore size of the cartilage matrix is approximately 6.0 nm [214], considerably smaller than most bacteria (100–4000 nm) or even bacterial spores (about 100 nm). Moreover, the negative charge of DNA further impedes its diffusion into cartilage, as only positively charged molecules readily accumulate within the negatively charged cartilage matrix; even neutral molecules exhibit limited mobility [215]. Since most bacterial cell walls, both Gram-positive and Gram-negative, also carry a net negative charge, their direct penetration is similarly hindered [215]. Therefore, bacterial penetration into healthy cartilage is improbable without significant tissue erosion. Persistent vascular channels may allow microbiota from the gut to reach cartilage, particularly through subchondral bone marrow, which may harbor latent bacteria. A study indicated that dysbiosis caused by pathogenic bacteria in subchondral marrow influences chondrocyte epigenetics [216].

Collectively, bacterial signals detected in joint tissues are unlikely to originate from direct infiltration. Instead, they may derive from viable bacteria that previously translocated to the subchondral bone marrow before cartilage breakdown, or from immune cells (such as macrophages) that phagocytosed bacteria at mucosal sites and subsequently migrated to the synovium. Further research is needed to clarify the exact origin and viability of these bacterial populations.

### Gut–joint axis

Results from the Rotterdam cohort of 1427 participants reveal that increased levels of *Streptococcus* spp. in the stool microbiome drive higher OA pain (WOMAC scores), independent of BMI [209], implying *Streptococcus* spp. may positively correlate with OA pain. Furthermore, converging evidence from murine models confirms the gut microbiota as a key modulator of OA pathogenesis. Germ-free mice show protection against post-traumatic OA with reduced severity in cartilage structure, osteophyte size, and synovial hyperplasia compared to specific pathogen-free (SPF) mice [217]. Similarly, the Murphy Roths Large (MRL)/MpJ “superhealer” mouse strain exhibits a 70% reduction in OA histopathology Osteoarthritis Research Society International (OARSI) score and significant decreases in synovitis and osteophyte scores post-DMM operation [218]. These findings emphasize the gut–joint axis’s role, with specific taxa like *Streptococcus* spp. implicated in OA pathology.

In 2025, Yang et al. identified a functional gut-joint axis in OA. Reduced microbial metabolite glycoursodeoxycholic acid (GUDCA) in OA patients correlates with OA severity, whereas inhibition of intestinal farnesoid X receptor (FXR), the receptor for GUDCA, alleviates OA by promoting intestinal L-cell proliferation and GLP-1 secretion. Moreover, OA patients have decreased abundance of *Clostridium bolteae*, a bacterium that promotes GUDCA precursor production, while supplementation with *C. bolteae* or the clinically approved drug ursodeoxycholic acid (UDCA) mitigates OA in mouse models via the gut FXR–GLP-1 axis [219].

Nevertheless, a growing body of literature indicates a strong connection between microbial dysbiosis and obesity-related OA [220, 221]. A 2015 preclinical study found that diet-induced obesity in rats caused joint damage linked to changes in microbial populations, with *Lactobacillus* spp. negatively correlating with *Methanobrevibacter* spp. positively correlating with OA severity [222]. Additionally, using Toll-like receptor-5 deficient (*Tlr5* KO) mice treated with antibiotics effectively prevented cartilage damage associated with obesity-induced OA [223]. Furthermore, obesity in mice causes a gut microbial shift, reducing beneficial *Bifidobacteria* and increasing harmful pro-inflammatory species, accelerating synovial inflammatory response. Restoring gut microbial community profile using oligofructose supplementation protects against DMM-induced OA of obesity by reducing synovitis and cartilage degeneration [224]. These results implicate gut microbial dysbiosis as a modifiable pathogenic factor that contributes to the aggravated OA phenotype in obese states.

### Gut microbiota dysbiosis-induced OA by LPS

A key way gut dysbiosis may contribute to OA is through the systemic translocation of microbial molecules, especially lipopolysaccharide (LPS). This “metabolic endotoxemia” involves bacterial products that escape the gastrointestinal tract into the bloodstream, leading to low-grade inflammation. In the blood, LPS interacts with LPS-binding protein (LBP), and then activates TLR4 on immune cells like macrophages to trigger inflammatory response involved in OA [225, 226]. Elevated levels of LPS and LBP in OA patients’ serum are associated with synovitis and increased KOA severity [227]. LPS, mainly generated by Gram-negative bacteria, LBP exhibits a broader recognition spectrum, binding pro-inflammatory elements from diverse pathogens including Gram-positive and Gram-negative bacteria, spirochetes, and mycobacteria [228, 229]. Moreover, the short half-life of LPS makes it technically challenging to measure, whereas LBP is a stable biomarker in circulation [230]. Consequently, both biomarkers could indicate microbial dysbiosis-related metabolic endotoxemia in OA, but LBP may be more effective in detecting gut-mediated inflammation contributing to OA pathology.

The extent to which LPS reduction can ameliorate OA is unknown, but clinical observations provide indirect support. First, in patients undergoing bariatric surgery for symptomatic OA, massive weight loss (20%) concurrently alleviated joint pain and reduced systemic inflammation [231, 232]. This implies a shared pathway linking metabolic health to OA. Furthermore, adipose tissue is now recognized as a key sensor of microbial dysbiosis [224]. Foundational research showed that introducing a conventional microbiota to germ-free mice triggered a 60% increase in body fat and insulin resistance, demonstrating the microbiome’s potent role in regulating adiposity [205]. In summary, the gut microbial community may modulate OA pathology by influencing body composition and low-grade inflammation, positioning adipose tissue as a crucial signaling hub in this gut–joint axis.

### Treatment of gut microbiota dysbiosis-associated OA

In recent years, gut microbiota-targeted interventions, including prebiotics, probiotics, and fecal microbiota transplantation, have gained recognition for managing OA [233]. Among these interventions, probiotics have been the most extensively studied, with the genus *Lactobacillus* at the forefront of research [234, 235]. For instance, *Lactobacillus acidophilus* has been proven to effectively reduce cartilage damage and inhibit the release of inflammatory factors [236]. Additionally, dietary modifications and prebiotics can restore microbial balance and lessen OA severity, with studies indicating that oligofructose supplementation in mice improves the abundance of beneficial bacteria and reduces inflammation associated with joint issues [224].

Fecal microbiota transplantation, which involves transferring fecal material from a healthy donor to a patient’s gastrointestinal tract, has shown effectiveness in treating inflammatory bowel disease [237, 238]. Emerging evidence also supports its potential in OA research and management after Huang et al. found that fecal samples from KOA patients with metabolic syndrome worsened OA severity in germ-free mice, indicating its potential in studying OA pathogenesis. However, the small sample size of this study (*n *= 9) prompts the need for further studies by recruiting large population of patients [239].

However, evidence for gut microbiota modulation remains primarily from preclinical animal models, with limited confirmation from large clinical trials. A notable randomized double-blind placebo-controlled trial of 537 OA patients found that 6-month supplementation with *Lactobacillus casei* Shirota (LcS) significantly improved WOMAC and Visual Analogue Scale (VAS) scores and reduced serum high-sensitivity C-reactive protein (hs-CRP) levels [240], supporting that LcS could be a novel anti-inflammatory strategy for managing OA.

**Figure 6.**
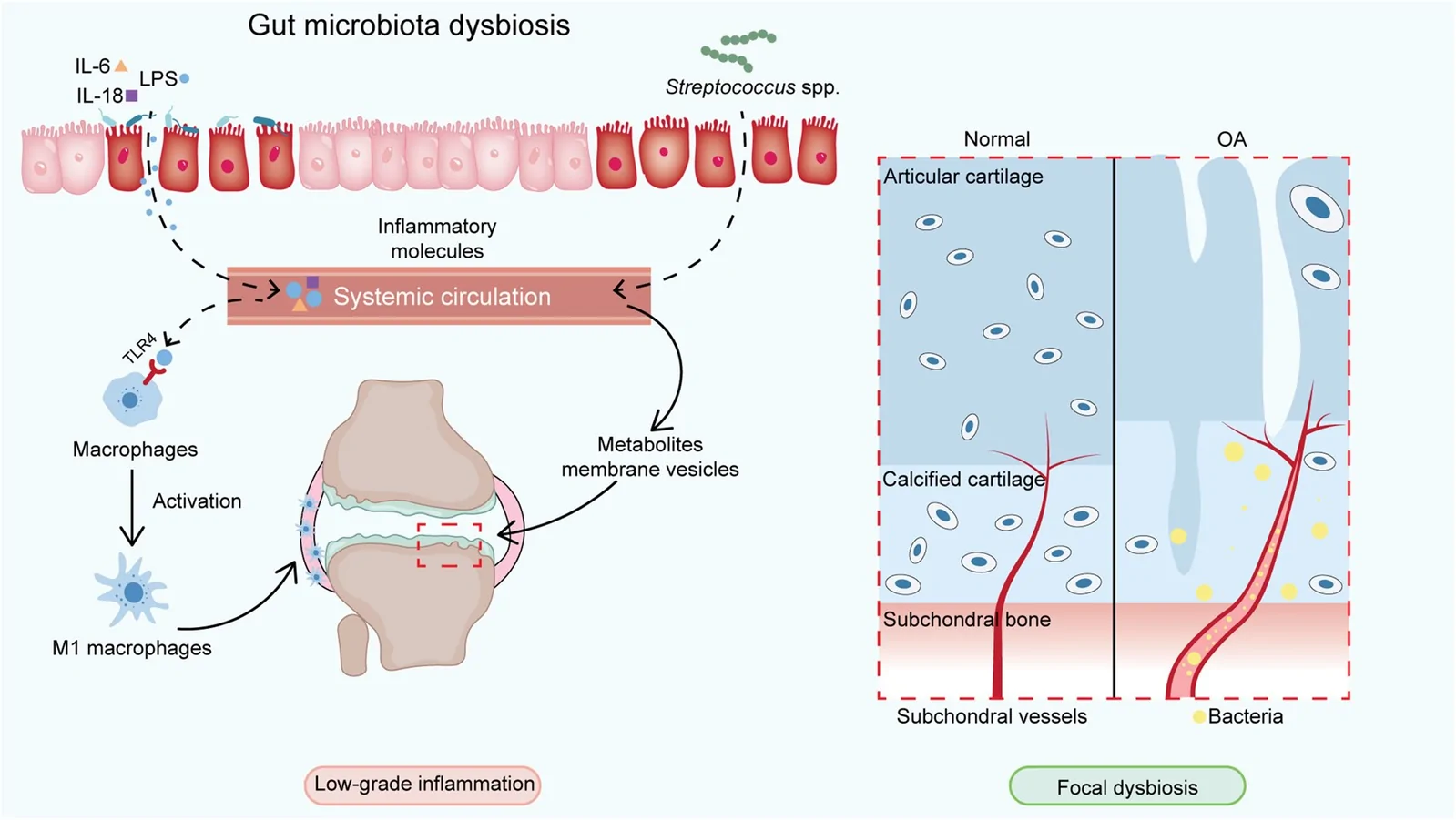
Schematic representation of the link between gut microbiota dysbiosis and osteoarthritis. Dysbiosis of the gut microbiota promotes the translocation of microbial products, including LPS and metabolites/membrane vesicles, into the bloodstream. Notably, greater *Streptococcus* spp. abundance results in more bacterial products in the circulation. These bacterial products cross the gut-blood barrier, either migrating directly to the knee joint to activate synovial macrophages, thereby causing local inflammation, or entering the circulation to polarize macrophages, thereby instigating a systemic inflammatory state that exacerbates joint damage. In the calcified zone of core regions, extensive areas of permanent cartilage are nourished by vascular channels. These channels become more numerous and dilated, potentially serving as a conduit for commensal bacteria to translocate from the bloodstream into the calcified cartilage and subchondral bone. Furthermore, breaches in the tidemark may permit bacterial ingress into the articular cartilage itself.

Thus, the gut-joint axis in OA operates mainly through LPS-induced systemic low-grade inflammation (Fig. 6). Modulation of the gut microbiome emerges as a potentially novel therapeutic candidate for OA management, particularly in obese individuals. Specifically, targeting the abundance of specific gut bacterial taxa (e.g., *Clostridium bolteae*, *Streptococcus* spp.) or regulating the related FXR signaling pathway can protect articular cartilage and delay OA progression. Further research is warranted to identify the specific bacteria and their derived substances that induce OA pathogenesis.

## Inflammation

Accumulating evidence indicates that components of metabolic diseases may contribute to low-grade inflammation during OA development. Obesity, as a major symptom of metabolic disorder, is a significant risk factor for OA, leading to chronic synovial inflammation [241]. Increased cholesterol levels increased synovial inflammation in early-stage collagenase-induced OA [145]. Moreover, under inflammatory conditions, increased LDL particles can be oxidized to oxLDL, activating synovial macrophages [144]. The uptake of oxLDL by LOX-1 in these macrophages induces an M1 polarization, activating the release of pro-inflammatory factors such as IL-1β, IL-6, and TNF-α, alongside chemotactic factors like CCL2 that promote monocyte recruitment, exacerbating inflammation. Additionally, synovial tissue in OA patients with T2DM displays higher TNF-α levels than those without diabetes [98]. Hypertension can cause vascular contraction, reducing blood flow to the synovium and triggering synovial macrophage pro-inflammatory polarization through hypoxia [168, 242]. These findings indicate that metabolic abnormalities are directly linked to local inflammatory responses by activation of synovial macrophages in OA joints.

Moreover, an increasing number of endogenous molecules termed damage-associated molecular patterns (DAMPs) are released by injured cells [243, 244]. The main types of DAMPs include high mobility group box protein 1 (HMGB1), members of the S100/calgranulin family (e.g., S100A8 and S100A9), and modified extracellular proteins (e.g., AGEs). Typically, under conditions of metabolic stress, DAMPs produced by damaged chondrocytes are released from the cell nucleus into the extracellular environment. These DAMPs are then detected by intracellular DAMP-sensing receptors such as TLRs and receptor for advanced glycation end products (RAGE) in synovial macrophages, activating an innate inflammatory response. This process, in fact, the initial induction of inflammation is protective which eliminates the damaged or dead cells; however, sustained inflammatory response exacerbates OA pathogenesis [226, 245]. Studies have shown that HMGB1, S100A8, and S100A9 can bind to TLR2 or TLR4, initiating inflammation through myeloid differentiation factor 88 (MyD88)-dependent signaling pathways. MyD88 then promotes recruitment and activation of mitogen-activated protein kinase (MAPK) and I-κB kinase (IKK). These activated kinases further drive the expression of inflammatory cytokines by activating transcription factors activator such as protein 1 (AP-1) and NF-κB [226, 245]. TLR4, a pivotal innate immune receptor, is required for HMGB1 to trigger TNF release in macrophages [246]. Upon activation, TLR4 signaling initiates downstream cascades that mobilize molecules like NF-κB and MAPK, enhancing the expression of pro-inflammatory cytokines. Accumulation of hyperglycemia-induced AGEs in chondrocytes is recognized by macrophage-expressed RAGE, leading to increased IL-6 production via the MAPK/NF-κB pathway [247]. Reports also indicate that HMGB1, AGEs, and S100A9 can increase RAGE expression, suggesting the existence of a positive feedback loop that amplifies RAGE-mediated inflammatory responses [248]. Moreover, macrophages themselves secrete HMGB1, S100A8, and S100A9 in response to inflammatory cytokines like IL-1β and TNF-α, creating a cycle that further amplifies the inflammatory cascade in OA joints.

**Figure 7.**
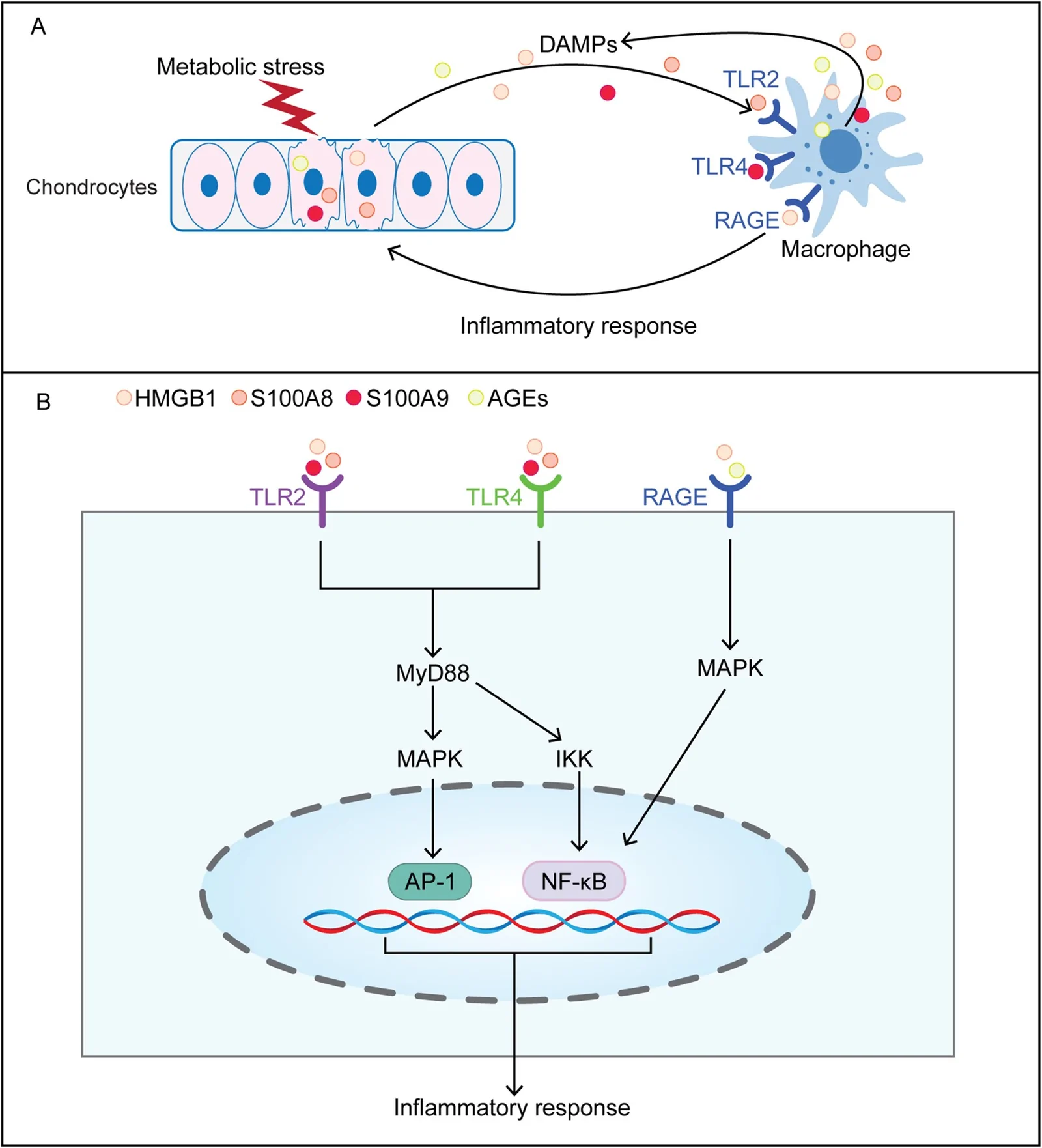
Intercellular DAMPs-induced inflammation in the pathogenesis of metabolic osteoarthritis. (A) Cartilage damaged by metabolic stress initiates the release of DAMPs including HMGB-1, AGEs, S100A8, and S100A9. Subsequently, DAMPs are recognized by DAMPs sensing receptors located on macrophages to initiate inflammatory responses and also secrete DAMPs. Elevated inflammatory levels exacerbate cartilage deterioration, resulting in the secretion of more DAMPs, thus creating a vicious cycle of inflammation. (B) These DAMPs are involved in various molecular pathways that lead to the transcription of inflammatory cytokines. HMGB1, S100A8, and S100A9 proteins associate with TLR2 and TLR4, triggering a signaling cascade through the MyD88 pathway, which enhances inflammatory responses through the activation of the transcription factors AP-1 and NF-κB, respectively. RAGE interacts with HMGB1 to facilitate inflammation via activation of NF-κB signaling. AGEs bind RAGE to activate downstream MAPK/NF-κB signaling pathway, leading to the subsequent production of IL-6.

Thus, metabolic stress fuels the flames of OA inflammatory processes by direct and indirect activation of macrophages via DAMPs sensing pathway. The innate immune players (DAMPs, DAMPs sensing receptors) and pro-inflammatory cytokines can mutually regulate each other between chondrocytes and macrophages, thereby exacerbating cartilage damage. Notably, multiple metabolic stressors, including lipid disorders, abnormalities of glucose metabolism and hypertension-induced hypoxia, converge onto shared central inflammatory pathways, such as NF-κB signaling, MAPK cascades, and TLR-MyD88-driven innate immune activation, all of which critically regulate macrophage polarization and sustained sterile inflammation (Fig. 7). This cascade establishes a self-perpetuating pathogenic inflammatory loop in metabolism-related OA. Therefore, targeting DAMPs to block sterile inflammation represents a promising therapeutic strategy. Future studies are necessary to understand how normalizing lipid and glucose metabolism can eliminate long-term “metabolic memory” and thereby resolve chronic inflammation in OA.

## Defects in mitochondrial function

Over the past decade, research has demonstrated that metabolic diseases can either trigger or worsen the OA phenotype through shared biological pathways like chronic inflammation. Additionally, mitochondrial dysfunction may link metabolic disorders to OA [249]. Mitochondria, commonly known as the “powerhouse of the cell,” are double-membrane organelles. They utilize energetic substrates (e.g., lipids and glucose) and oxygen to generate adenosine triphosphate (ATP) via the inner membranes of the electron transport chain (ETC) [250, 251]. Mitochondria, beyond generating energy, are the primary source of ROS. During ATP synthesis, they release less than 5% of the consumed oxygen as reactive oxygen species(ROS), which are neutralized by mitochondrial antioxidants, keeping ROS levels normal [252]. Disruption of the mitochondrial function results in elevated intracellular ROS, which can induce oxidative stress, characterized by an imbalance between ROS generation and degradation [253].

Articular chondrocytes contain a certain amount of mitochondria and produce ROS [254]. Previous study identified a correlation between the overproduction of ROS resulting from mitochondrial dysfunction and the onset of ageing-related OA [255]. Currently, pathological changes with mitochondrial dysfunction in chondrocytes make significant contributions to the OA progression, particularly when triggered by high glucose concentration [100], AGEs [256], hypercholesterolemia [134] or elevated expression of adipokines such as leptin [257]. For example, the accumulation of cholesterol particularly cholesterol oxidation products (oxysterols) in mitochondria can disrupt complexes of the electron transport chain. This disruption leads to the overproduction of mitochondrial ROS, ultimately inducing oxidative stress [258]. Moreover, high glucose concentrations can enhance the flux of the tricarboxylic acid (TCA) cycle in mitochondria, increasing the production of reducing hydrogen and creating a proton concentration gradient. This results in an excess of high-energy electrons binding with oxygen, forming oxygen-free radicals and ultimately causing overproduction of ROS [259], indicating that a high-glucose environment raises ROS levels.

Increased levels of ROS caused by metabolic factors contribute to OA progression through multiple mechanisms such as suppressed matrix synthesis, MMP activation, cytokine induction, and the induction of chondrocyte apoptosis [260, 261]. Critically, ROS impair mitochondrial DNA and its repair, creating a cycle that further boosts ROS production and drives apoptotic cell death [254]. Reed et al. confirmed that ROS directly mediates mitochondrial dysfunction and promotes matrix degradation via oxidation of ECM components [262]. Moreover, in OA chondrocytes with high glucose environments, continuous glycosylation reactions produce AGEs, which not only disrupt the cartilage matrix structure by forming cross-linked complexes between proteins, but also activate NF-κB, Bcl-2-associated X protein (Bax), nicotinamide adenine dinucleotide phosphate (NADPH) oxidase through RAGE phosphorylation, increasing the production of superoxide anions in chondrocytes [263]. AGEs also induced mitochondrial dysfunction of chondrocytes by inhibiting the AMPK/SIRT1/peroxisome proliferator-activated receptor γ coactivator 1 (PGC-1) signaling pathway [256]. This connection between mitochondrial integrity and OA is further supported by emerging evidence linking gut microbiota composition to mitochondrial dysfunction in OA models [219]. Interventions with probiotics or prebiotics demonstrate therapeutic potential by rebalancing the gut microbiome, enhancing mitochondrial performance, reducing inflammatory markers, and attenuating cartilage destruction [264]. The expression of LOX-1, an ox-LDL receptor in chondrocytes, is promoted by IL-1β and ox-LDL itself. Ligand binding to LOX-1 stimulates ROS production, thereby activating the NF-κB pathway [265]. Once activated, NF-κB drives a pro-inflammatory state within OA tissues, upregulating the expression of mediators including inducible inducible nitric oxide synthase (iNOS), IL-8, and COX-2 [266]. Thus, mitochondrial dysfunction is intricately linked to inflammatory processes in OA.

AMPK is closely related to mitochondrial oxidative stress, functioning not only as a sensor for redox signals, but also has anti-oxidant properties once activated [267]. AMPK activators, metformin and berberine have been demonstrated to have chondroprotective effect on inhibition of OA progression [112, 268]. More importantly, metformin has recently been shown to effectively relieve OA symptoms in RCT studies in patients with obesity-associated OA, highlighting its clinical potential as a promising therapeutic candidate for obesity related OA [269].

Beyond AMPK, the Sirtuin (SIRT) family of NAD^+^-dependent deacetylases represents another critical layer of regulators in OA pathophysiology. Reduced SIRT activity is detrimental to cartilage homeostasis [267]. Diabetic OA patients showed lower SIRT2 expression, higher ROS levels and more severe inflammation. Functional studies further confirmed that inhibiting SIRT2 accelerates OA progression in diabetic patients, while upregulating SIRT2 alleviates OA development. This protective effect of SIRT2 is mediated by suppressing excessive oxidative stress and inflammatory reactions [270].

**Figure 8.**
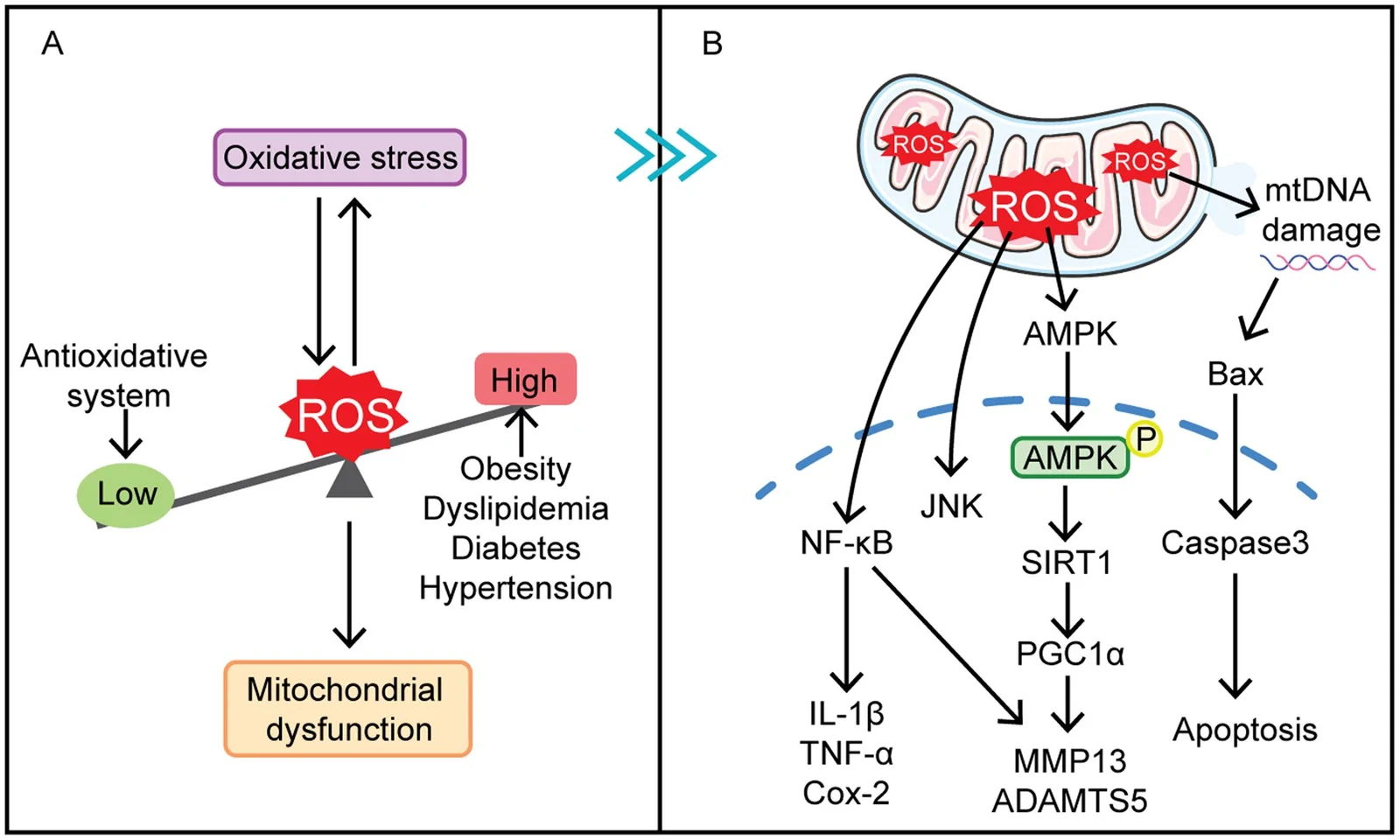
Proposed mechanisms for chondrocyte mitochondrial dysfunction in metabolic disease-linked OA. (A) Metabolic stressors trigger mitochondrial dysfunction in chondrocytes, leading to excessive ROS production that overwhelms the antioxidative system and induces oxidative stress. (B) This process is accompanied by mtDNA damage. Elevated ROS directly oxidize ECM components and activate transcription factors, promoting the expression of pro-inflammatory mediators (IL-1β, TNF-α, and COX-2) and matrix-degrading enzymes (MMP13 and ADAMTS5), thereby accelerating cartilage catabolism. Concurrently, mitochondrial dysfunction suppresses the AMPK/SIRT1/PGC-1α signaling axis, further compromising cellular stress adaptation. ROS and metabolic insults also activate pro-apoptotic pathways, culminating in chondrocyte apoptosis and joint degeneration.

Direct targeting of ROS also confers chondroprotection. Inhibition of ROS prevents cartilage loss by suppressing oxidative stress as well as catabolic proteases [271]. Additionally, ROS scavengers slow cartilage loss in OA animal models with joint inflammation and reduce the expression of MMPs in chondrocytes (Fig. 8) [272].

Thus, metabolic disturbances may cause oxidative stress-induced mitochondrial dysfunction in OA joints due to high levels of ROS. ROS provides a “non-energetic” intracellular signal that activates numerous transcription factors, including NF-κB, JNK, and MAPK, promoting ECM degradation, chondrocyte apoptosis, and increased cytokine-induced chondrocyte inflammation. Drugs that lower ROS or activate AMPK signaling, such as metformin, may help limit cartilage damage, suggesting that targeting mitochondrial dysfunction could be a promising therapeutic strategy for OA progression.

## Conclusion and future perspective

We provide an overview of the current understanding of the molecular mechanisms in metabolic abnormalities-associated OA. Obesity directly contributes to OA by increasing mechanical stress on weight-bearing joints and indirectly through systemic and local inflammation mediated by adipokines and adipose tissue macrophages. In the pathogenesis of diabetic OA, the accumulation of AGEs accelerates cartilage breakdown, whereas hyperglycemia-induced synovitis is exacerbated by the crosstalk between FLSs and macrophages. Dyslipidemia causes ectopic lipid deposition in chondrocytes and synovial macrophages, leading to cartilage degradation and the activation of pro-inflammatory mediators. The existence of endothelial–skeletal interaction in the joints emphasizes the potential role of systemic factors such as RAS, endothelins, and Wnt signaling in hypertension-induced OA. Specifically, the LPS produced from gut bacteria worsens localized joint injury via systemic metabolic endotoxemia.

In order to address metabolic OA, new etiology-targeted pharmaceutical therapies are being considered for OA management. To facilitate the reduction of adiposity in obese patients with OA, alongside exercise, medications such as thiazolidinediones (TZDs), which exert off-target effects with specificity for peroxisome proliferator-activated receptor gamma (PPARγ). Moreover, dual glucose-dependent insulinotropic polypeptide (GIP)/GLP-1 agonists, such as tirzepatide, have recently emerged as powerful candidates for metabolic OA therapy, particularly in patients with obesity and T2DM [273]. Furthermore, well-designed, controlled trials for these novel applications of conventional drugs are on the horizon and it is time to bring in a new era of personalized prophylaxis for OA.
